# Time-trend and variations in the proportion of second-eye cataract surgery

**DOI:** 10.1186/1472-6963-7-53

**Published:** 2007-04-13

**Authors:** Lorena Hoffmeister, Rubén Román, Mercè Comas, Francesc Cots, Enrique Bernal-Delgado, Xavier Castells

**Affiliations:** 1 Evaluation and Clinical Epidemiology Department, Institut Municipal d'Assistència Sanitària (IMAS), Passeig Marítim, 25-29, 08003, Barcelona, Spain; 2 Health Services Research Department, Instituto Aragonés de Ciencias de la Salud, Gómez Laguna 25, 50009, Zaragoza, Spain

## Abstract

**Background:**

Despite recommendations for greater use of second-eye cataract surgery and the bilateral progression of the disease, there is a substantial proportion of unmet need for this treatment. Few studies have explored the factors associated with second-eye cataract surgery utilisation. The objective of our study was to estimate the proportion of second-eye cataract surgery, evaluate its time-trend, and explore differences in utilisation by patients' gender, age, and region of residence.

**Methods:**

All senile cataract surgeries performed between 1999 and 2002 in the public health system of Catalonia (Spain) were obtained from the Minimum Data Set. The proportion of second-eye surgery from November 2000 to December 2002 was calculated. The time-trend of this proportion was characterised through linear regression models with the logarithmic transformation of time.

**Results:**

The proportion of second-eye surgery was 30.0% and showed an increasing trend from 24.8% (95% Confidence Interval [CI] 21.6; 26.1) in November 2000 to 31.8% (95% CI 31.4; 33.6) in December 2002. This proportion was 1.9% (95% CI 0.9; 2.9) higher in women (p < 0.001) and held constant across time. Male patients aged less than 60 had the lowest proportion (22.6%; 95% CI 22.4; 22.9) and females between 70 and 79 had the highest proportion (27.4%; 95% CI 26.9; 27.9). The time-trend for the proportion of second-eye surgery in those aged over 80 years was greater than for younger ages, showing an increase of 9% at the end of the period for both males and females. Variations between regions decreased over time because regions with the lowest initial proportions of second-eye surgery (approximately 17%) showed a greater increase over the study period.

**Conclusion:**

We predict greater utilization of second-eye surgery in patients aged 70 to 79 years and in women. A greater increase in the utilisation rates of second-eye surgery is expected in the regions with lower proportions and in older patients. The observed trend suggests that there will be a substantial proportion of unmet need for bilateral surgery.

## Background

In the last few decades, cataract surgery rates have markedly increased in Western countries [1-4]. A substantial part of this increase is due to the increasing number of patients undergoing surgery in both eyes [4-6], that is, they have a second-eye cataract surgery after their first surgery. Several studies have demonstrated the benefit of second-eye surgery especially in stereopsis and in patient-reported visual disability[5,7-13]. Bilateral cataracts are usually removed one eye at a time, mainly due to the risk of major complications such as endophthalmitis. Some studies conclude that the benefits of second-eye surgery are greater when the time interval between surgeries is reduced[11,14].

Few studies have explored the factors associated with second-eye cataract surgery utilisation [15-17]. Gender differences have been reported to be more pronounced in second-eye surgery than in first-eye surgery across all age groups[6,17]. A greater age at operation has also been pointed out. According to Lundstrom et al[6], the increase in second-eye surgery rates has resulted in an increase of surgeries performed in the elderly.

Several studies have shown variations in the overall rate of cataract surgery within and among countries. These rates varied from 3.8 to 41.2 per 1,000 Medicare beneficiaries in distinct geographic areas in the USA[18,19] and from 16.7 to 61.8 per 10,000 inhabitants in the United Kingdom[20]. In Catalonia[21], the cataract surgery rate in 1993 was 21.9 per 10,000 inhabitants, with a ratio of 4.5 between the highest and lowest surgery rates in the 26 areas analysed. To date, the presence of geographic variations in second-eye cataract surgery utilisation and their influence on overall rates has not been studied.

In the United Kingdom, the proportion of second-eye surgery was 35% of all cataract surgeries performed in 1997[15]. In Sweden[4], a proportion of 36.8% was found in 1999, presenting an increasing tendency between 1992 and 1999. In the USA[5] and in Spain[16], during a 12-month follow-up, a quarter of the surgeries performed were second-eye surgeries. Despite recommendations for greater use of second-eye cataract surgery and the bilateral progression of the disease, there is a substantial proportion of unmet need for this treatment[16].

In this context of increasing unmet need for cataract surgery[22] and greater utilisation [1-4], the volume of second-eye surgery is important. The causes are the pressure on costs[5], the still important proportion of people who die before undergoing surgery despite the increase of surgery rates[22], and the management of waiting lists in the public health system, as prioritization systems are usually based on the visual acuity of both eyes, thus giving less priority to patients waiting for second-eye surgery [23-25].

The aim of this study was to estimate, over the total number of cataract surgeries in the public health system, the proportion of second-eye cataract surgery, evaluate its time-trend, and explore differences in utilisation by patients' gender, age, and region of residence.

## Methods

### Setting and patients

**Figure 1:**
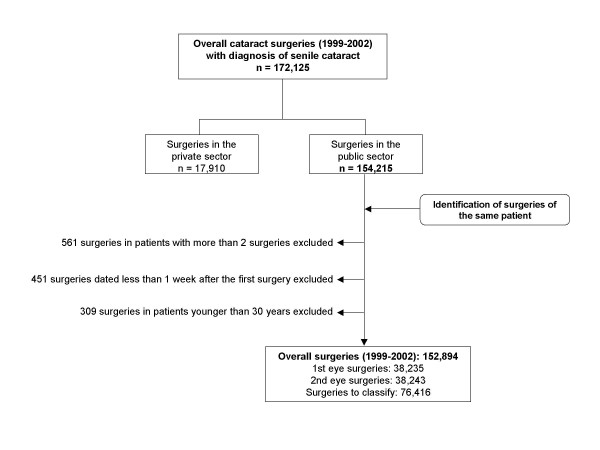
Flow diagram of data included in the analysis.

Information was obtained from the Minimum Data Set of Catalonia, which includes 172,125 episodes of cataract surgery performed between January 1999 and December 2002 (figure 1). The study period was chosen to be representative of the current cataract surgery rate: since 1999, the use of phacoemulsification in the outpatient setting has been widespread in Catalonia, and second-eye surgery has become routine. Privately financed cataract surgeries (10.4%) were excluded, resulting in 154,215 surgeries in public hospitals. Cataract surgery procedures (codes 13.1 to 13.59 and 13.71 of the ICD-9-CM) performed for senile cataract (codes 366.10 to 366.19, 366.8 and 366.9 of the ICD-9-CM) were included.

The information for each procedure included the patient's gender, age at surgery, and region of residence. Age at surgery was grouped into four categories: less than 60 years, between 60 and 69 years, between 70 and 79 years, and 80 years or older. Catalonia (6,343,110 inhabitants according to the 2001 census) was divided into seven health regions. Of the total number of surgeries, 1.5% (2,279) could not be assigned to a particular region and were excluded from the analysis of regions only.

The study followed the tenets of the Declaration of Helsinki and was approved by the ethical committee of the research centre.

### Calculation of the proportion of second-eye surgery

The Minimum Data Set does not record whether a cataract procedure was a first- or second-eye operation. For the 154,215 public sector cataract surgeries included in the study (figure 1), surgeries in the same patient were identified through their blinded clinical record number and hospital identifier (corresponding to 115,104 patients). To reduce possible biases in the calculations due to errors in the registry, 561 surgeries of 185 patients with more than two recorded surgeries were excluded, as well as 451 surgeries dated less than 1 week after the first-eye surgery (not considered as different) and 309 surgeries in patients younger than 30 years (not considered as senile, figure 1).

Between 1999 and 2002, one-third of the patients (about 38,200) underwent surgery in both eyes (figure 1). In the remaining 76,416 patients who underwent one surgery only in this period, it was necessary to establish whether the surgery corresponded to first-eye surgery without a second surgery before December 2002 or second-eye surgery with the first eye being operated on prior to January 1999. In order to classify these surgeries, the time interval between the first and second surgery in patients who underwent two surgeries in the study period was calculated. The mean time interval was 7.5 months with a median of 4.9 months and 95% of the patients underwent second-eye surgery 22 months or less after first-eye surgery. The 95^th ^percentile was considered as the maximum time interval between the two surgeries, allowing errors in the identification of first- and second-eye surgeries to be reduced to 5%. Thus, the surgeries performed in the first 22 months (between January 1999 and October 2000) were used only to identify, when appropriate, the corresponding second-eye surgery performed from November 2000 onwards, while the unilateral surgeries performed after that date were considered as first-eye surgeries.

### Analysis

The proportion of second-eye surgery was calculated for each month between November 2000 and December 2002 as number of second-eye surgeries divided by overall number of surgeries per month. The age and gender standardised rates in each region were calculated for both first- and second-eye surgery by direct standardisation to the 2001 population.

A linear regression model was adjusted with the observed monthly proportion of second-eye surgery as the dependent variable. This model included the natural logarithmic transformation of time in months (ranging from 1 to 26) as the independent variable because there was a linear relationship between the proportion of second-eye surgery and the log-transformation of time. Thus, the observed proportion of second-eye surgery was characterised throughout the study period. This proportion showed a logarithmic shape, starting with a pronounced increase but showing moderate growth at the end of the period. Although the most appropriate model for proportions was the logistic model, the adjusted linear model provided similar predicted values and better goodness-of-fit statistics. For the sake of clarity in the interpretation of results, the linear regression model was used.

Two multivariate models were developed. One assessed differences in the proportion of second-eye surgery according to age and gender and the other assessed differences among regions. Both models included the logarithmic transformation of time, the coefficient(s) associated with the factors under study, and the interaction among them as independent variables.

## Results

Between November 2000 and December 2002, 67,197 first-eye and 28,860 second-eye surgeries were performed. The mean age was 74.4 years at first-eye surgery and 75.0 years at second-eye surgery, while 58.5% of first-eye surgeries and 60.9% of second-eye surgeries were performed in women.

**Figure 2:**
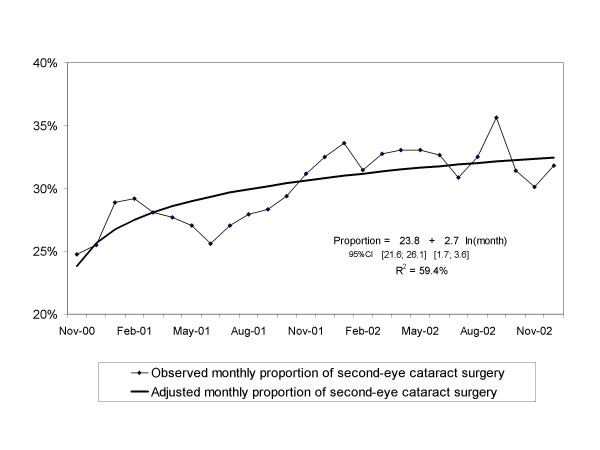
Observed and adjusted value of the proportion of second-eye cataract surgery in Catalonia.

In the study period, the overall proportion of second-eye surgery was 30.0%. An increasing tendency in the proportion of second-eye surgery over time was observed (figure 2). Table 1 shows the proportion of second-eye surgery stratified by gender, age, and region. The highest proportions were found in women (31.1%, 95% CI 30.7; 31.5) and in patients aged 70 to 79 (31.4%, 95% CI 31.0; 31.8). Differences among regions ranged from a proportion of 25.5% (95% CI 24.5; 26.5) in Girona to 33.6% (95% CI 32.8; 34.5) in Barcelonès Nord-Maresme.

**Table 1:** Descriptive proportions of second-eye surgery between November 2000 and December 2002

	**Proportion**	**95% Confidence Interval**
Proportion of second-eye surgery in women	31.1	30.7; 31.5
Proportion of second-eye surgery in men	29.0	28.6; 29.5
Proportion of second-eye surgery by age groups		
Less than 60 years	27.0	25.9; 28.0
60 – 69 years	30.4	29.7; 31.0
70 – 79 years	31.4	31.0; 31.8
80 and older	28.1	27.5; 28.6
Proportion of second-eye surgery by region		
Lleida	26.9	25.9; 28.1
Tarragona-Terres de l'Ebre	25.6	24.5; 26.8
Girona	25.5	24.5; 26.5
Costa de Ponent	28.1	27.5; 28.9
Barcelonès Nord-Maresme	33.6	32.8; 34.5
Centre	32.8	32.2; 33.5
Barcelona Ciutat	30.9	30.4; 31.5

Residuals of the regression models were checked and normality was accepted for all of them (data not shown). The overall regression model (not including age, gender and region) showed a statistically significant increasing tendency and explained 59.4% of the variance in the proportions observed (figure 2). At the end of the study period, the adjusted proportion was 32.5% (95% CI 31.4; 33.6). Projecting to a 5-year time horizon showed that, in 2007, 35.7% (95% CI 33.6; 37.7) of cataract surgery would be performed in second eyes.

**Figure 3:**
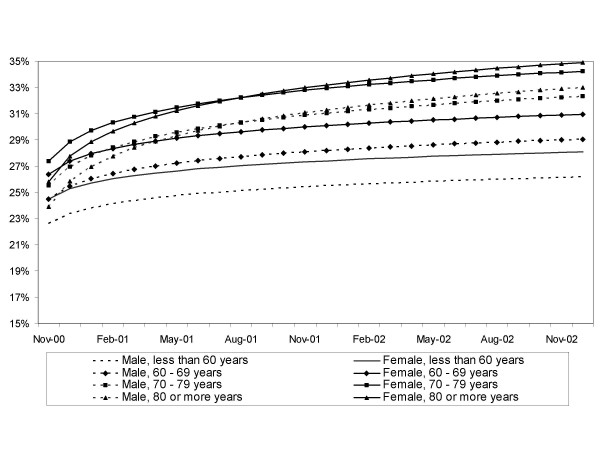
Adjusted value of the proportion of second-eye cataract surgery by age and gender.

The regression model including gender and age showed that the effect of this two factors on the proportion of second-eye surgery was independent since no interaction between age and gender, or between age, gender and time was found. The proportion of second-eye surgery was 1.9% (95% CI 0.9%; 2.9%) greater for women (p < 0.001) than for men. This difference held constant across time since no significant interaction between gender and time was found (p = 0.441). Male patients aged less than 60 had the lowest proportion in oposition to females aged between 70 and 79 which had the highest proportion (figure 3). In all age groups the proportion of second-eye surgery significantly increased during the study period, this increase was greater as age increased (figure 3). Patients over 80 years of age had the highest increase in the proportion of second-eye surgery, reaching 32.9% (95% CI 31.3%; 34.5%) for males and 34.8% (95% CI 33.2%; 36.4%) for females at the end of the period studied (December 2002). Patients aged less than 60 years had the smallest increase reaching a proportion of 26.4% (95% CI 24.8%; 28.0%) for males and 28.3% (95% CI 26.7%; 29.9%) for females at the end of the period (figure 3).

**Figure 4:**
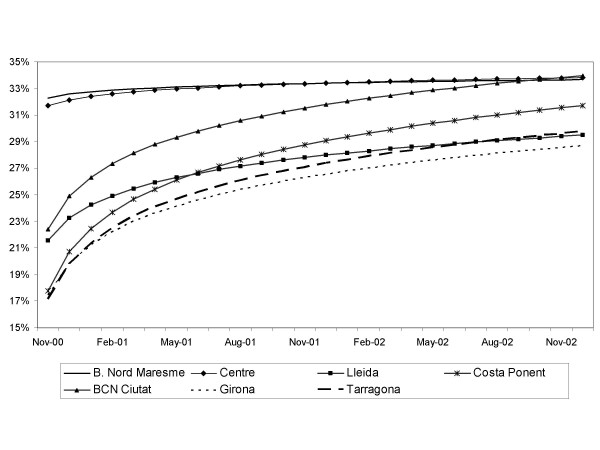
Adjusted value of the proportion of second-eye cataract surgery by region.

Significant differences among health regions were found in the proportion of second-eye surgery when the logarithmic model including regions was adjusted (figure 4). The highest proportions were found in Barcelonès Nord-Maresme and Centre (approximately 32%), followed by Lleida and the City of Barcelona, which showed intermediate proportions. Finally, the remaining three regions had proportions of around 17%. The time trend showed that regions with the lowest proportion at the beginning of the study period (November 2000) had a significantly greater increasing tendency of around 4% (figure 4).

Variations in the overall rate of cataract surgery (first- and second-eye surgery) among regions were found. In 2001, the overall rate was 190.5 surgeries per 10,000 inhabitants aged 50 years or older and the rate of second-eye surgery was 55.3 surgeries per 10,000 inhabitants aged 50 years or older. Variation in age-gender standardised cataract surgery rates among regions ranged from 126.4 in Tarragona-Terres de l'Ebre to 238.4 in Barcelonès Nord-Maresme. Variations in the standardised rates of second-eye surgery ranged from 29.5 to 78.9 surgeries, corresponding to a ratio of 2.7, while those for first-eye surgery ranged from 97.2 to 159.4, corresponding to a ratio of 1.6.

**Figure 5:**
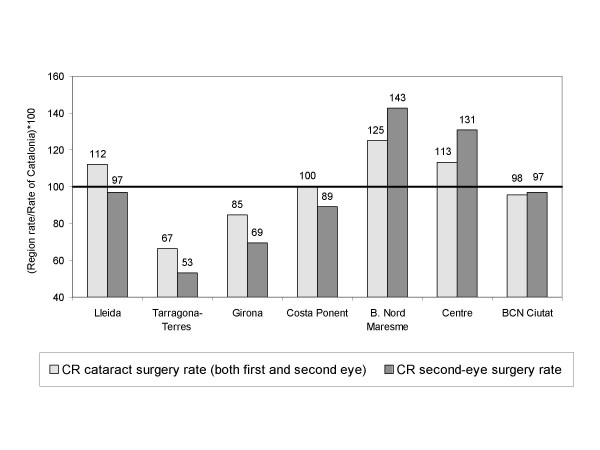
**Comparative Ratio of the age-sex standardised overall cataract surgery rate and second-eye surgery rate, 2001**. The first bar represents the comparative ratio (CR) between the overall surgery rate, standardised by age-sex, for each region with respect to the overall rate in Catalonia. The second bar represents the second-eye surgery rate for each region with respect to the overall second-eye surgery rate in Catalonia.

Figure 5 shows the relationship between variations in the overall cataract surgery rate and variations in second-eye surgeries. In regions with the highest and lowest cataract surgery rates, variations with respect to the overall rate were mainly due to differences in second-eye surgery rates. Barcelonès Nord-Maresme and Centre, which showed the highest utilisation of cataract surgery, had a higher use of second-eye surgery.

## Discussion

During the study period, 30% of cataract surgeries performed in the public system corresponded to second-eye surgery. The proportion found showed an increasing tendency. The extent and tendency of second-eye surgery were similar to those found in studies performed in the United Kingdom[15] and Sweden[4].

Bearing in mind the bilateral progression of cataracts and the evidence of the effectiveness of second-eye surgery[5,7-13], the maximum theoretical limit of the proportion of second-eye surgery would be 50%, which would mean that all patients would undergo surgery on both eyes. However, Castells[8] pointed out that not all patients would benefit equally from second-eye surgery; some patients show general deterioration that would make them unsuitable for a second-surgery[6]. Therefore, a proportion of somewhat less than 50% can be expected. In Sweden, the projected volume of second-eye surgery is approximately 45%[6]. Thus, despite the increasing trend in the proportion of second-eye surgery observed in Catalonia and a projected proportion of around 36% in 2007, a substantial proportion of patients will probably not undergo this surgery. This information on unmet needs in cataract surgery is useful not only to health managers, but also to ophthalmologists.

The gender differences found in the present study agree with previous reports showing higher overall rates of cataract surgery and of second-eye surgery rates in women[6,17]. Although one of the most plausible explanations for this finding has been greater survival in women, our results suggest that other factors (such as preferences or comorbidity) have a greater influence than survival, as no interaction between age and gender was found.

The overall rate of cataract surgery is higher in older age groups[2-4,6]. Nevertheless, some studies[6] have found a flattening or decline in the utilisation of this surgery among the very old (patients aged around 80 or 85 years old) and a lower willingness to refer elderly patients for cataract surgery[26]. In agreement with the findings of our study, Castells *et al*.[16] found that older patients had a lower probability of undergoing bilateral surgery. Our data show greater growth among the oldest patients, progressively reducing differences in bilateral cataract surgery utilisation among age groups. This tendency could be explained by broadening the indications for cataract surgery among the very old and by evidence showing that these patients derive as much benefit from second-eye surgery as younger patients[8]. Another factor is the tendency to shorten the time interval between first- and second-eye cataract surgery in the elderly[14] to increase the length of time a patient can benefit from bilateral surgery.

To our knowledge, the present study is the first to explore geographical variations in the proportion of second-eye surgery. The differences found among regions were not unexpected, especially in view of the wide variability in the overall rates of cataract surgery [18-21]. We expected a greater variation in second-eye cataract surgery utilisation and consequently, that this variations would contribute importantly to the differences observed in the global rate of cataract surgeries among regions. Because the benefit of second-eye cataract surgery is lower than that of first eye surgery, the decision made by the patient and the clinician is more influenced by factors other than patient preference (e.g.: accessibility or need perception). The influence of these factors in the context of the Spanish health system should be studied in future studies. However, throughout the study period, differences among regions diminished, with a more pronounced increase in the regions with lower initial proportions. The marginal increase in some regions might be due to high initial utilisation, close to the level at which the proportion of second-eye surgery settles (36%).

Lleida, Girona and Tarragona are the regions with the largest rural population and showed a marked increase, although these regions had the lowest proportions of second-eye surgery at the end of the period. The greatest increase in the proportion of second-eye surgeries was observed for the city of Barcelona (the capital and most populated city) and Costa de Ponent, which is adjacent to the city Barcelona. Barcelonès Nord-Maresme and Centre, which showed a steady, but high, proportion of second-eye surgeries, are regions with a substantial population density, which is mostly urban, and and are also adjacent to the city of Barcelona.

Several models were checked to adjust the tendency over time, including logistic regression and time-series analysis. The most appropriate option for adjusting the observed growth curve through time was the log-transformation of time because there was a linear relationship between the proportion of second-eye surgery and the log-transformation of time. Although a multivariate model including all three factors and time and the interactions among them was considered to be the most appropriate, the number of surgeries for some combinations of factors was too small to allow confident estimation due to the smaller number of surgeries in some regions. Thus, the analysis by regions was separated from the analysis by age and gender.

Another key point is the limitation to identify as first- or second-eye surgeries those surgeries of patients having only one surgery within the 4-year period. The threshold of 22 months was chosen in order to minimize missclassification errors, which would underestimate the proportion of second-eye surgeries for the months close to the threshold, and to allow sufficient time window to analyze time-trends with minimal influence of missclassification errors. The threshold was calculated for each region and little variation was found (data not shown).

The MDS of Catalonia is an exhaustive and systematic registry with mandatory inclusion of all surgical procedures performed in the public health system. This registry shows negligible magnitudes of missing values, with only 0.02% of cataract surgeries without the patient's gender and 0.03% without the patient's age at surgery, while only 0.16% of the total number of patients presented more than two cataract surgeries. To guarantee accurate identification of the number of surgeries per person, various forms of aggregation were tested and all provided similar proportions of second-eye cataract surgery (data not shown). One of the limitations of this study was the impossibility of identifying patients undergoing surgery in different hospitals, either in the public or in the private sector. However, some studies suggest that most patients undergo surgery in the same hospital[16,27]. Thus, the proportion of second-eye surgeries would not be seriously underestimated.

## Conclusion

In conclusion, in Catalonia, the proportion of second-eye surgery is currently increasing. A reduction in variations among regions and age groups was observed (although not in gender differences), with the most pronounced growth among the oldest age groups and regions with lower utilisation of second-eye surgery. If the interval between surgeries is reduced, the proportion of second-eye surgeries will probably rise substantially, thus increasing the tension between supply, unmet need, and waiting list management. Likewise, the results of this study reveal the need to balance the greater accessibility to first-eye surgery to the elderly on the one hand and the enhanced benefit of bilateral cataract surgery to individuals on the other.

## Competing interests

The author(s) declare that they have no competing interests.

## Authors' contributions

All the authors have contributed to the achievement of this study. LH performed the main statistical analysis, participated in the design of the study, and drafted the manuscript. RR and MC participated in the design of the study, the statistical analysis and drafting of the manuscript. FC and EB participated in the design of the study and reviewed the manuscript. XC conceived of the study, and participated in its design and coordination and helped to draft and review the manuscript. All authors read and approved the final manuscript.

## Pre-publication history

The pre-publication history for this paper can be accessed here:


